# ‘The last thing you feel is the self‐disgust’. The role of self‐directed disgust in men who have attempted suicide: A grounded theory study

**DOI:** 10.1111/papt.12389

**Published:** 2022-02-28

**Authors:** David Mason, Darren James, Liz Andrew, John R. E. Fox

**Affiliations:** ^1^ 2112 Cardiff University Cardiff UK; ^2^ Aneurin Bevan Health Board Newport UK

**Keywords:** attempted suicide, emotion, grounded theory, male, qualitative, self‐disgust

## Abstract

**Objective:**

Globally, suicide affects more men than women. Emotional pain underpins many theoretical accounts of suicidality, yet little is known about the role of disgust in suicide. Self‐directed disgust, whereby aspects of the self‐serve as an object of disgust, has been hypothesised to factor in suicide. This research aimed to explore the processes which link self‐disgust to attempted suicide in males.

**Method:**

Nine men who had attempted suicide completed semi‐structured interviews. The interview data were analysed using a constructivist grounded theory methodology.

**Results:**

Three concepts emerged out of the analysis: (1) self‐disgust; (2) worthlessness; and (3) the endured emotional distress of ‘the abyss’” – these concepts interweaved, leading the men to experience hopelessness, disconnection and an inability to cope, leading ultimately to their suicide attempt. Throughout this journey, various disgust‐related processes worsened men's distress and increased their suicidal risk. Historic adversities prevailed across the data, as did the men's difficulties in understanding their emotions.

**Conclusion:**

Self‐disgust was an important emotion in the men's experiences of suicide and shaped their views of themselves and their lives. The distancing and repellent properties of self‐disgust, in addition to the fear of having their ‘disgustingness’ exposed, increased suicidal risk. Self‐disgust appeared more pervasive in the suicidality of men with a history of multiple childhood adversities. The limitations of this research are discussed as implications for clinical practice and directions for future research.

**Practitioner points**

- Self‐disgust is a pervasive emotion in men's experience of attempted suicide.
- Self‐disgust appears marked in men with a history of multiple early adversities.
- This paper provides a novel understanding of self‐disgust in male suicide, which is missing in the literature.

## INTRODUCTION

It is estimated that over 800,000 people die from suicide each year (World Health Organization; WHO, 2014). The past two decades has seen an increase in deaths by suicide in the United States, with males at a greater risk of death by suicide (Hedegaard et al., 2020). In 2018, the United Kingdom recorded just over 6500 deaths by suicide (Simms et al., 2019). There are well‐established gender differences in suicidality, with men at a three times greater risk of death by suicide (WHO, 2014). For each death by suicide, it is estimated that there are 20–30 individuals who attempt suicide (Wasserman, 2016).

Psychological models have attempted to conceptualise the aetiology and multifactorial nature of suicide risk, including static and dynamic risk factors (for a comprehensive review of models of suicidality, see Barzilay & Apter, 2014 and O’Connor & Nock, 2014). Conceptualisations of suicide are limited in their ability to explain gender differences in suicide, as males are much more likely to die from suicide (Canetto, 2017; Canetto & Sakinofsky, 1998; Möller‐Leimkühler, 2003). Hegemonic masculinity is regularly cited in gender studies literature as cultural and societal rules and scripts which legitimise men's dominant social position (for a history of the term, see Connell & Messerschmidt, 2005). Cleary’s (2005, 2012) study into male suicide in Ireland found that men who have attempted suicide were more likely to report hegemonic masculinity and that led to reduced help seeking, the men's concealment of distress and their usage of more maladaptive coping strategies (Cleary, 2005, 2012). This pattern mirrors male responses to mental health difficulties (Krumm et al., 2017; McKenzie et al., 2016).

Psychological pain and hopelessness are common across many conceptualisations of suicide and are synonymous with emotional pain (Williams, 1997). Suicide has often been conceptualised as an escape from this psychological pain (e.g., Baumeister, 1990; Shneidman, 1993; Williams, 1997). This pain has been defined as meta‐emotional experiences of sadness, anxiety, fear, weakness, rejection, loneliness, emptiness, worthlessness and shame (Berglund et al., 2016; Lakeman & Fitzgerald, 2008; Lester, 1997). Gender differences in emotional expressiveness are well documented (see Ashfield & Gouws, 2019). It is possible that gender differences in emotional expression and management are likely to not only impact mental health, but also underpin the disproportionately high rate of male suicide relative to females (Canetto, 2017; Canetto & Sakinofsky, 1998; Payne et al., 2008). Crucially, higher levels of anger, guilt, self‐directed hostility and shame have been found to increase suicide risk (Rogers et al., 2017) and it could be argued that increased hostility, shame and guilt could significantly impact males due to their reluctance to express emotions and seek appropriate help and support (Möller‐Leimkühler, 2003; Payne et al., 2008).

The increased levels of shame and guilt implicated in suicide highlight the potential role of disgust, as shame and guilt have been considered as disgust's secondary emotions (Power & Dalgleish, 2016). To date, the empirical literature has neglected the role of disgust in suicide. Yet, it has been hypothesised to factor in mental health conditions associated with a high risk of suicide, including depression (Phillips et al., 1998) and eating disorders (Fox & Power, 2009; Olatunji & McKay, 2009).

Disgust's adaptive role helps to avoid physical and moral contamination and is defined by a physical response to push away substances which are perceived as noxious or repugnant (Olatunji & McKay, 2009; Rozin & Fallon, 1987). Self‐disgust describes the maladaptive internalisation of the disgust response. Here, the disgust eliciting object is seen as oneself and one's behaviours and results in a negative disgust‐based self‐evaluation (Powell et al., 2015a). Self‐disgust is conceptualised as a distinct emotional schema (Izard, 2007, 2009) and is differentiated from other subjective emotional states such as self‐hatred, embarrassment, guilt and shame (Powell et al., 2015a). It is focused around feelings of contamination and repulsion, visceral physiological experiences of nausea and specific disgust‐based cognitions such as ‘I’m revolting’ (Powell et al., 2015a). Further differentiation is present in the behavioural correlates of disgust, including attempts to avoid contamination and ‘extreme attempts to cleanse or remove the disgusting self’ (Clarke et al., 2019, p. 111). Therefore, hypothetically, it follows that self‐disgust may factor in suicide as an extreme attempt to eliminate a contaminated sense of self. Empirical support for this proposition comes from self‐disgust's location in mental health difficulties (Clarke et al., 2019; Powell et al., 2015b), including conditions associated with high suicide risk, such as eating disorders (Chu et al., 2015; Fox, 2009; Fox & Power, 2009), post‐traumatic stress disorder (PTSD; Brake et al., 2017) and depression (Overton et al., 2008; Powell et al., 2013, 2014; Simpson et al., 2010). Chu et al. (2013) situates self‐disgust within the interpersonal theory of suicide (Joiner, 2005; Van Orden et al., 2010) by proposing that suicidality increases by generating feelings of inadequacy and a perceived burdensomeness. Interestingly, given male tendency to withdraw when struggling with mental health difficulties, Chu et al. (2013) argue that disgust becomes directed towards others and the world which, in turn, leads to a perception of the world as contaminated, resulting in distancing, isolation and a thwarted belongingness. Although research linking self‐disgust and suicide is in its infancy, Brake et al. (2017) found that self‐disgust significantly moderated the effect between PTSD symptoms and suicidal ideation.

### The current study

Power and Dalgleish (2016) describe self‐disgust as a ‘major component [of suicide] that has failed to be investigated’ (p. 323). This study aims to bridge this gap by exploring the process of self‐disgust in men who have attempted suicide. An inductive qualitative methodology is well suited to explore this research aim and qualitative approaches have been welcomed in suicide research (Hjelmeland & Knizek, 2010). Grounded theory is one such method which provides a framework to identify categories of data and to integrate them into theory describing emergent processes (Willig, 2013). Qualitative research has situated self‐disgust in females (Powell et al., 2014); however, it has yet to be investigated in a male only sample. Furthermore, quantitative research on self‐disgust often reports a majority female sample (e.g., Brake et al., 2017; Chu et al., 2015; Overton et al., 2008; Powell et al., 2013; Simpson et al., 2010). As described above, men may have different emotional experiences to women and due to their increased risk of death by suicide; it therefore seems pertinent to explore self‐disgust within a male only population. It remains an empirical question as to whether particular self‐disgust concepts are more pertinent for men. However, despite the predominantly female samples used across the extant self‐disgust literature, mixed‐gendered research identifying self‐disgust across mental health conditions associated with suicide (e.g., depression, self‐harm, PTSD) has not reported gender differences (for a review, see Clarke et al., 2019). This suggests that self‐disgust could be a factor in male psychological distress and suicide, which warrants further research scrutiny. Furthermore, it could be hypothesised that self‐disgust's repellent and distancing properties may factor in suicidal men's self‐isolation, which is a significant risk factor in male suicide (Oliffe et al., 2019).

The study presented here has two aims.

1. To determine the degree to which men identify self‐disgust as an important emotion within their suicide attempts.
2. To understand the potential processes that link self‐disgust to attempted suicide for males.

## METHOD

### Recruitment of participants

Ethical approval was obtained from a local research ethics committee. Participants were recruited from NHS secondary care community mental health teams (CMHT) and third sector organisations. Interested individuals were directed to a Qualtrics survey (Qualtrics XM, Provo, UT) and potential participants were contacted by the researcher for further telephone screening. The inclusion and exclusion criteria are described in Table 1. There is no precedence in the literature regarding recruiting timeframes for individuals who have attempted to die by suicide. In order to ensure that interviews were carried out safely, there was a requirement of a six‐month interval between participants’ last suicide attempt and the interview date. This was to provide time for suicidality to decrease and allow for crisis support to be accessed (if needed). Despite the emotive topic of study, research indicates that qualitative interviews exploring suicidality do not increase suicide risk and appear to be beneficial (Biddle et al., 2013; Reynolds et al., 2006).

**TABLE 1:** Study inclusion and exclusion criteria

Inclusion criteria	Exclusion criteria
Male Eighteen and over Fluent in English Had made a previous suicide attempt over six‐months ago Currently open to a CMHT with a named health care professional (CMHT recruitment only) Consenting to provide contact details, including GP, in order to manage any potential risk	Any current suicidality (i.e. suicidal ideation, thoughts, ideas or plans) Any current self‐harm behaviour or ideation Currently open to crisis/home treatment teams Individuals with organic brain disorders Individuals whose suicide attempt was in the context of a psychotic episode Individuals (and healthcare professionals) who believed that taking part may increase suicidality or self‐harm risk

Twenty people self‐selected to participate (11 from CMHTs and 9 from the third sector). Eleven individuals were excluded from the study; see Table 2 for reasons for not taking part in the study. The remaining nine participants (seven from CMHTs and two from the third sector) met the inclusion criteria and took part in the study.

**TABLE 2:** Reasons for non‐inclusion in the study

Reasons for not taking part in the study	
Did not agree to provide details of address or GP	*N* = 3
Did not respond to research correspondence	*N* = 3
Reported a recent deterioration in mental health	*N* = 2
Felt an interview could increase risk	*N* = 1
Did not have an allocated healthcare professional	*N* = 1
Suicide attempt was in the context of a psychotic episode	*N* = 1

Note

(Please note, any prospective participants reporting mental health concerns were signposted to appropriate support).

### Participants

Participants were residents of the United kingdom and were aged between 24 and 54 (mean 39.89, standard deviation =10.25). All identified as White‐British. The majority of participants were heterosexual (*n* = 7), with the remainder identifying as homosexual (*n* = 1) or not disclosed (*n* = 1). Seven of the men had accessed psychological support, whereas two had never accessed mental health services. The sample characteristics are shown in Table 3.

**TABLE 3:** Overview of participants

Name (pseudonym)	Age	Relationship status	Mental health diagnoses	Duration since last attempt	Most recent attempt method	Number of prior attempts	Self‐Disgust Scale – Revised (SDS‐R; Powell et al., 2015c)		
							Physical	Behavioural	Total
Huw	39	Divorced/Separated	EUPD Depression Anxiety	10 years ago	Overdose	3	22	23	70
Ian	39	Single	EUPD	2 years ago	Overdose/Self‐harm	Over 4	31	22	71
Jack	52	Married	Paranoia	4 years ago	Deliberate vehicle crash	2	18	17	57
Jacob	48	Divorced/Separated	EUPD	2 years ago	Overdose	1	27	26	75
Lewis	24	Single	EUPD Depression Anxiety	18–24 months ago	Attempted drowning	1	29	14	67
Luke	54	Married	PTSD	12–18 months ago	Attempted jumping	3	35	19	85
Rhys	39	Single	BPAD Depression	6–12 months ago	Overdose	3	21	14	45
Richard	37	Divorced/ Separated	Anxiety Depression Mania	6–12 months ago	Hanging	3	30	20	76
Tom	27	Married	None	6–12 months ago	Hanging	0	21	17	52

Abbreviations: BPAD, Bi‐polar affective disorder; EUPD, Emotionally unstable personality disorder; PTSD, Post‐traumatic stress disorder.

### Data collection and procedure

#### Measures

##### Demographic questionnaire

This questionnaire recorded basic demographic data, such as age, ethnicity, sexuality, relationship status and mental health diagnoses.

Suicide questionnaire (Appendix A) – This questionnaire was created by the authors to situate the sample regarding participant's suicidality. The questionnaire recorded data around participant's suicidal behaviours, including timeframe, method, subjective intention, writing of suicide notes and whether help was sought (either medical or personal). The questionnaire also asked the number of historic suicide attempts.

##### Self‐Disgust Scale Revised (SDS‐R; Powell et al., 2015c)

The SDS‐R is a validated, 22‐item measure of self‐disgust scored on a seven‐point Likert scale. Participants rate their agreement with a statement about the self, ranging from 1 (strongly disagree) to 7 (strongly agree). It provides a physical and a behavioural self‐disgust score ranging from 5 to 35 and a total self‐disgust score ranging from 15 to 105. Higher scores indicate higher levels of self‐disgust.

#### Data collection

Data were collected by face‐to‐face semi‐structured interviews, which provide flexibility and allow for follow‐up prompts (Barker et al., 2016). Interviews lasted between 60 and 90 min which included a debrief for each participant that assessed risk. The interviews were audio recorded and transcribed verbatim.

### Interview schedule

A semi‐structured interview covered the participant's suicidality, their understanding of self‐disgust and their experiences of self‐disgust during episodes of suicidality. It explored the development and context of individual's suicidality and questions sought to explore participant's emotional experiences during suicidality, especially selfdisgust. The final interview schedule can be found in Appendix B.

### Data analysis

Interview data were analysed using the principles of grounded theory (Glaser & Strauss, 1967) and using Nvivo Software (QSR International, Version 12). Grounded theory focusses on processes, patterns and meaning within a particular context and aims to explore relationships between different concepts. A constructivist approach of grounded theory was used (Charmaz, 2014), which views any findings as a construction of the researcher's understanding of the phenomenon of interest (Willig, 2013). Grounded theory was chosen over other qualitative methods, such as Interpretative Phenomenological Analysis (IPA), as the research aims focused more on theoretical questions relating to self‐disgust and individual's suicide attempts. IPA does not provide these insights to the same degree as grounded theory, as it is much more focused on the experience of a phenomena. Grounded theory data collection and analysis run in parallel with each other. The process of data analysis involved coding, categorisation and theory development, with memo writing used throughout to explore analytical ideas while remaining grounded in the data (Charmaz, 2014). Each transcript was initially coded line‐by‐line for the action, experience, process and meaning being conveyed by the participant. This was followed by focused coding, which involved raising the most pertinent initial codes that make the most analytic sense of the whole data (Sbaraini et al., 2011). Focused codes which help to explain the processes occurring in data were then raised (or merged) into conceptual categories and descriptive concepts which were used to explain the whole data (Birks & Mills, 2011; Willig, 2013). Theoretical coding was used to explain relationships between categories, codes and concepts. Constant comparison and memo writing were used throughout the research process. In keeping with a grounded theory methodology, the interview schedule was adapted multiple times as ideas and concepts emerged during the early data analysis.

#### Methods to enhance quality and reflexivity

A constructivist paradigm views the researcher's role as ‘actively constructing’ theory, rather than solely capturing it (Willig, 2013, p. 80). The lead researchers in this study are both men who are mental health professionals with a history of working with suicidal men. Both are qualitative researchers, with (initials removed) having previously published qualitative research, including grounded theories. A range of methods were used to enhance quality and reflexivity throughout the research project (i.e., Ahern, 1999; Elliott et al., 1999), including peer review with an independent, female, qualitative researcher.

Service‐user input was provided into the research design to improve the relevance, quality and sensitivity of the project (Staniszewska et al., 2017). This input involved the development of the research question and the usage of the three questionnaires. Service‐user input also informed the language and content of the research materials (including readability), guidance on the interview schedule, and the potential impact of a qualitative interview on self‐disgust and suicide. This input also assisted and signposted the research team to possible recruitment centres (within the third sector). Service‐user input helped to develop sensitive and comprehensive research documentation and design the interview process to increase participant's feelings of safety and agency. As a result, service‐user input facilitated a more sensitive and comfortable research experience for the participants.

## RESULTS

**FIGURE 1:**
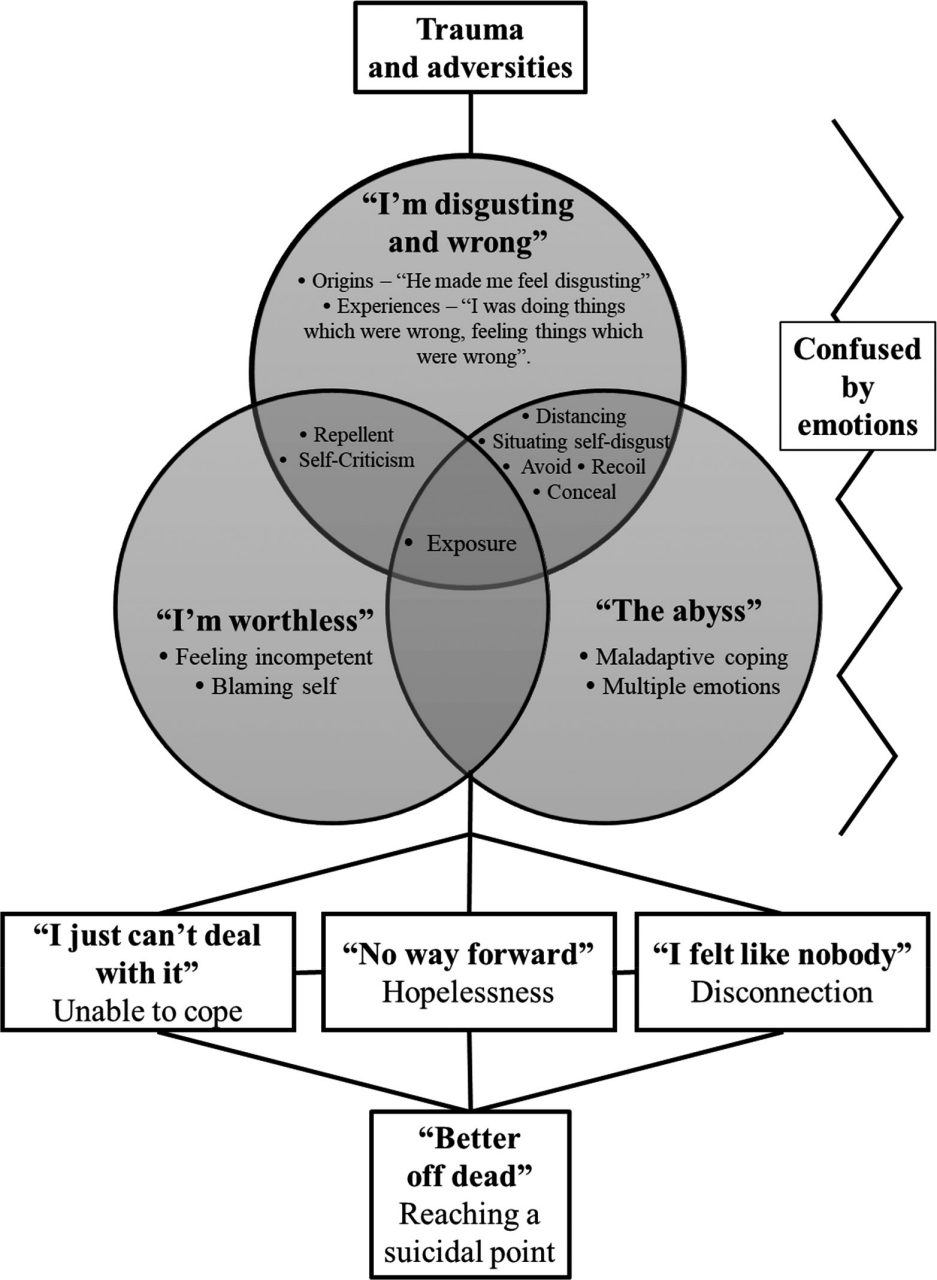
Grounded theory representation of men's journey to attempted suicide

Self‐disgust was an important emotion within men's suicidality and was linked to their suicide attempts. Self‐disgust did not explain the men's attempts in their entirety, instead it interweaved with other factors to increase suicide risk. Participants’ journeys to suicide began at an early age, with all participants describing a history of early *trauma and adversity*. These early experiences provided a context for distress, encompassing participants’ sense of self as ‘*disgusting and wrong’*, *‘worthless’* and in an endured emotional ‘*abyss’*. Various disgust processes (e.g., distancing) within these concepts worsened distress and increased suicidality. A risk of having one's ‘disgustingness’ *exposed* was a key process in exacerbating suicide risk. The men situated their distress within a history of being *confused by emotions* which had the potential to intensify their pain. This position left the men feeling *unable to cope*, *disconnected* and *hopeless*, which ultimately led them to *reach a suicidal point*. From here, suicide was perceived as a solution to their distress and led to their attempt. The intensity and positioning of self‐disgust varied for the men at different points during this journey and appeared more pervasive for participants with a history of multiple adversities. Through constant comparison methods, a representation of this journey, which is grounded in the participants’ accounts, is shown in Figure 1. Each category and concept is described below with illustrative quotes. Quotations were chosen for their comprehensiveness, while also safeguarding anonymity. Pseudonyms are used throughout.

### Trauma and adversities

Early trauma was reported by participants, including childhood physical, sexual and emotional abuse, neglect and bullying. The majority of participants had experienced multiple early adversities. All participants highlighted the distress of these early experiences and how it attributed to them feeling ‘*disgusting and wrong’*, *‘worthless’*, in an endured emotional ‘*abyss’* and *confused by emotions*.

Those situations in my childhood has affected my view on life and led me to feel this way. (Tom)

### ‘I’m disgusting and wrong’ (Ian)

All participants described a lived experience of self‐disgust which punctuated their lives at different points and with diverse consequences. This concept comprises of two categories which relate to the *origins* and *experience* of the ‘disgusting’ self.

#### Origins: ‘He [father] made me feel disgusting’ (Jacob)

The majority of participants recalled experiencing self‐disgust from an early age. Many participants perceived their ‘disgustingness’ to originate from either their *trauma and adversities* or from an awareness that their identity was incongruent with dominant social norms, therefore risking stigmatisation. For one individual, this related to his identity as a sexual minority.

A lot of my problems are definitely programmed by my parents’ attitude towards me, in the sense that I’m a bad person and therefore I’m disgusting and wrong, you know, which they instilled in me. (Ian)

They said it [homosexuality] wasn’t normal back then, you know. It was only something weird people did… I think as a child if your parent says something or a teacher says something or a policeman says something… you think they’re right. You think that they know better, because they do, generally… and so, if it was a disgusting thing to do, and a teacher told me… I’m sure that that’s why I think that way. (Huw)

Subjective experience: ‘I was doing things which were wrong, feeling things which were wrong.’ (Huw)

While some participants experienced self‐disgust as a briefer reactionary emotional state, most men commented on its enduring properties. Participants with more childhood adversities commented on the consuming qualities of self‐disgust, with childhood abuse appearing linked to a pervasive sense of the self being ‘disgusting’.

I think, sometimes, if you’ve got that level of disgust with myself like I have, it’s always in the back of your mind, it never goes away… That’s a lot to carry round with you. (Luke)

Self‐disgust was experienced in various ways. This included feeling disgusted about appearance, suicidal thought processes, mental health difficulties and their behaviours, including their achievements and treatment of themselves and others. Those with a greater number of adversities appeared to position disgust on more aspects of the self. Those who had been bullied tended to direct disgust more towards their image.

The disgust I feel now though, is that I will never‐, I will never be able to have a relationship, because other than the, the fact that, like I said, I’m big and ugly, I, I’ve lived so long‐, I’ve been told things are wrong. (Huw)

One participant provided a nuanced experience of self‐disgust related to childhood sexual abuse. He felt polluted by his experiences, feeling unclean and malodourous and feared the *exposure* of this, leading to avoidance. He describes *disconnection* from his younger abused self, which may be a strategy to manage the distress of this profound level of self‐disgust.

I constantly think I smell. I constantly think [pause and exhale] I’m dirty, disgusting [pause 7 seconds] … It makes me feel physically sick, when I think about him [younger abused self]… I hate him. (Luke)

There were further links between the processes of *exposure*, *disconnection* and participant's disgust, often involving mirrors. A number of participants experienced self‐disgust when exposed to their reflected self. Feeling disconnected from this reflection was a common experience, with some participants describing their reflection as ‘abnormal’ and ‘inhuman’ while experiencing visceral disgust responses. As stated above, this may have aided managing high initial levels of distress; however, it further perpetuated self‐disgust and worsened overall distress.

Sometimes I look in the mirror… when I’m in a really low point and I think ‘what is this thing looking back at me?’ I almost feel sick to the point, because it’s just, that’s my low state of mind at that point, when I’m feeling really low, I’m feeling depressed, you feel that cramp in your stomach and it feels like I’m about to throw up or something. (Lewis)

### Worsening emotional distress – ‘The abyss’ (Tom)

The ‘abyss’ was synonymous with participant's descriptions of their worsening and enduring distress. The abyss was permeated by the coexistence of multiple affect experiences including anger, sadness, fear, shame, embarrassment, guilt, self‐hatred and feeling weak and vulnerable. These perpetuated the abyss, as did multiple accumulating triggers, including relational difficulties and a perceived lack of support. The consequence of the abyss appeared to move men closer to *hopelessness* and ultimately to a *suicidal point*.

It just went from one thing to another. Erm, I, you know, it went from feeling sad, to feeling angry, to feeling frustrated, to feeling worthless to feeling, just that there wasn’t any point in me being there. It, it, each day was getting worse and worse and worse and it progressively got worse until that very night. (Lewis)

All participants navigated the abyss using maladaptive coping strategies. These included self‐medication with substance use, using food to regulate mood and self‐harm. The men's coping strategies perpetuated their distress, with some direct feedback loops which maintained self‐disgust.

I hate the way that I look and I know that part of the way that I look is due to the unhealthy stuff that I eat, and just eat something and then feel disgusting about what I’m eating, but before I know it I’ve got a chocolate bar in my hand, trying to make, pick myself up. (Richard)

#### Interactive processes between the abyss and ‘I’m disgusting and wrong’

Participant's attempts to cope with feelings of self‐disgust often interacted to worsen the abyss and exacerbate feelings of *disconnection*. Participants avoided, recoiled, concealed or distanced themselves from situations which may *expose* a distressing self‐disgust experience. Mirrors and other people were commonly avoided. Concealment was a further strategy to avoid *exposure*. However, two participants noted that rules around masculinity also caused them to conceal distress. Participants attempted to distance themselves from disgust by cleaning or changing parts of the self which were felt to be ‘disgusting’. This appeared to be more pertinent for those with a history of multiple traumas.

It’s [self‐disgust] horrible. That’s what makes you lock yourself away. You don’t want to be seen. (Rhys)

#### Situating self‐disgust in the abyss

All participants situated self‐disgust within their meta‐emotional experiences of the abyss. Self‐disgust appeared to work on a continuum and for some participants it was positioned closer to *a suicidal point*. For some men, this was exacerbated by *exposure*.

When you get to that point of looking in the mirror. It’s like, that’s like the last thing you feel is the self‐disgust, is like when you’ve already gone, you’ve had like months of going down. And you hit, like you hit the bottom and then you start having them feelings. (Rhys)

There was a general difficulty in differentiating self‐disgust from often coexisting emotional experiences such as self‐hatred, shame and embarrassment. Despite the difficulties in differentiating these emotions, it does highlight the salience of self‐disgust within the abyss. Participants who felt more able to delineate their meta‐emotional experiences differentiated self‐disgust by its the visceral properties, particularly nausea. Self‐disgust, shame and self‐hatred were situated closer than any other subjective experiences, particularly self‐disgust and self‐hatred. Some men highlighted disgust's repellent properties and intensity of dislike when differentiating self‐disgust from self‐hatred. This was noticeable in the lived experience of the interview, in which agitation arose when exploring self‐disgust. After a break, the participant reported his agitation to have decreased and wished to continue.

I’m finding that I’m getting agitated, cos I’m incredibly uncomfortable… about that… and it is, it’s not just hatred, it is, yeah, it is disgust. (Jacob)

### ‘I’m worthless’ (Lewis)

All participants resonated with the subjective experience of worthlessness, although these were more pronounced in participants with a history of bullying and childhood physical abuse. Judgements of worthlessness permeated the men's view of themselves, their relationships and their worth in society. Common to this were experiences that their suicide would be ‘better for others’. This moved the men towards *hopelessness* and experiencing *disconnection*, thereby increasing suicidal risk. Worthlessness was perpetuated by feelings of incompetence and self‐blame, leading the men to a sense of self as being ‘pathetic’ or a ‘failure’. This was more marked in individuals with a history of being bullied.

Yeah, I think I just felt like a bad father and a bad kind of husband and like a bad friend and a bad housemate and it just, like, just this worthless feeling… I dunno. It seems like no matter what I do I never feel worth. (Richard)

#### Interactive processes between ‘I’m worthless’ and ‘I’m disgusting and wrong’

Occasionally, participants described how the same attribute could trigger feelings of worthlessness and self‐disgust. However, most participants described the origins of self‐disgust occurring prior to their experiences of worthlessness. Therefore, a sense of worthlessness appeared to be a consequence of feeling ‘*disgusting and wrong’*. The process of self‐criticism perpetuated worthlessness and suicidal risk. However, the language used in participants’ self‐critical remarks highlighted the relationship between self‐disgust and worthlessness. For example, participants used expressions of wrong, dirt and contamination, such as ‘dudd’ (Tom), ‘crap’ (Jacob) and remarked on their perceived ‘disgusting’ appearance.

I just don’t think very much of me at times, at those times that I, I feel like scum. (Jacob)

### Exposure – ‘What if people find out what happened to me?’ (Jack)

As described above, the exposure of one's perceived ‘disgustingness’ perpetuated distress and self‐disgust. However, exposure had the potential to weave across all three concepts and increase suicide risk.

Interviewer – And what did that make you feel [experiencing flashbacks]?

Weak. Disgusting… I never feel clean… what happened to me shouldn’t happen to anybody. And it just makes me feel worthless, shit, I shouldn’t be here. (Luke)

Participants feared their perceived ‘disgustingness’ being exposed, including their mental health difficulties, suicidality, sexuality or childhood sexual abuse. One individual concealed his abuse as he feared its *exposure* would be emasculating. This highlights a potential nuanced relationship between sexual abuse, shame, disgust and masculinity.

It [trauma flashbacks and self‐disgust] pushes what’s in the back of your mind, it pushes to the front. I get the feeling of I don’t contribute nothing to the family…It makes you feel you’d be better off dead. I mean, no more trouble for anybody. (Luke)

### Confused by emotions – ‘You're talking to somebody who's never been able to label his emotions’ (Jacob)

Men's suicidality was increased due to perceiving themselves as ‘*disgusting’*, ‘*worthless’* and in an’*abyss’*. Far from being isolated concepts, they interacted with each other to worsen distress through the processes of *exposure*, avoidance, recoiling, concealment, distancing, repelling and self‐criticism. Across the data, it was apparent that this distress took place within the context of the men being *confused by their emotions*, which is indicative of alexithymia. When reflecting on their suicidality, the participants remembered struggling to place a name on their distress or understand their psychological processes. This suggests alexithymia tended to exacerbate the distress of the abyss and feelings of *disconnection* and *hopelessness*. Some men described difficulties in identifying emotional experiences during heightened distress, whereas others described a permanent inability to recognise emotions.

It was a constant mix of emotions which was really difficult to pinpoint if it was sadness, anger, regret, denial or whatever, like…It was just totally confusing. (Tom)

### Disconnection – ‘I felt like nobody just sat around. You just watch the world go by’ (Rhys)

As distress worsened, all the participants experienced disconnection from themselves, others and the world. Disconnection moved men closer to suicide either directly or indirectly by interacting with other experiences, such as *hopelessness*. Interpersonal disconnection exacerbated feelings of loneliness and further worsened distress.

I was lost out there in the world on my own, I thought nobody cared, I didn’t know who to turn to, you know. (Jack)

Disconnection could be a consequence of participants’ attempts to cope with self‐disgust and suggestive of the emotion's ‘pushing‐away’ or repelling function. Participants disconnected through recoiling, concealing, distancing and avoidance. For one participant, this directly preceded his *suicidal point*. Furthermore, some participants saw themselves as repellent, causing them to feel disconnected from others.

People start noticing I’m not doing anything, I’m not going out. And when I get to the point where you just, you really, you can’t answer the phone, you can’t answer the door, you can’t look out the windo… you don’t want anyone to look in your window. (Rhys)

What people recoil at, what people don’t like, the behaviours that I have are quite abhorrent, you know. (Jacob)

### Inability to cope – ‘I just can't deal with it’ (Tom)

While not endorsed by all participants, some men perceived an inability to cope with their distress as they *reached their suicidal point*. This was often exacerbated by maladaptive coping strategies. Often this state of helplessness was intertwined with experiences of *hopelessness*.

Just that you can’t cope with life. Or you feel like you’re not coping as well as other people. And, you dread that, you believe that everything’s just going to get worse, you know. You just don’t see any future. (Rhys)

### Hopelessness – ‘No way forward’

All participants moved towards a sense of hopelessness as they approached their suicide attempts. Hopelessness was associated with dejection, the perception of a bleak future and no alleviation to their suffering.

It’s just like. It’s the giving up on yourself, the hate on yourself and you just can’t see no way forward. And that’s what makes you, or made me, do it anyway. I just felt there’s no way this is going to get any better. (Rhys)

Figure 1 conceptualises how *hopelessness*, *disconnection* and *feeling unable to cope* can provide the context for a suicide attempt. These processes could work in isolation or in combination to increase suicide risk.

It’s when things stopped working, your coping strategies… you think that you run out of resources that are available to you, there is no way out, it’s not going to get better. I felt shit for having mental health problems, to add on top of everything. And unlovable. (Jacob)

### Approaching a suicidal point –’Better off dead’ (Luke)

The men's distress, feelings of *disconnection* and *hopelessness* and a perceived *inability to cope* led the participants to a position where suicide felt like the only available option. The majority of participants referred to the word ‘point’, suggestive of a junction between enduring distress, or a solution through suicide. Occasionally, triggering points (e.g., relational difficulties) acted as a catalyst to move the men towards suicide, although they were not always present. What was common was a cumulative effect of the previously discussed categories and concepts resulting in the participants’ suicide attempts.

[Step one] Self‐harming’s not helping. Step two; That feeling of total… even though you’re with somebody you love more than anything, it can be the loneliest place in the fucking world when you’re sat there. And if I get to there, that means I’m on step three [suicide attempt]. (Luke)

All participants positioned self‐disgust as a factor in their suicide attempt and four men placed it as a central component. Self‐disgust's positioning during the attempt was more prominent for participants with a history of multiple adversities.

The two times that I’ve done that [attempted suicide] is the times that I’m really properly thinking that’s disgusting behaviour, that I’m disgusting, because good people don’t do that. (Jacob)

For two individuals with multiple adversities, the *exposure* of their ‘disgustingness’ instantly triggered their suicide attempt. All of Luke's suicide attempts were in the context of experiencing trauma flashbacks. Additionally, Huw described an intentional overdose directly after his sexuality was exposed.

They came into the room and started calling me faggot and things like that [trauma details redacted]… and I just couldn’t cope…I bought lots of pills. (Huw)

A smaller number of participants described excessive use of mirrors when they approached their suicidal point. Routinely, these men had avoided mirrors and this suggests an intentional exposure to their ‘disgustingness’. During this exposure, the men witnessed their self‐harm and preparations to die, often while *disconnected*. It could be that the men were punishing themselves by facing the exposed, ‘disgusting‐self’, although one participant attributed his increased mirror usage to ‘saying goodbye’.

I watch myself putting the cigarettes out on my forehead… or taking the razorblade across my face… an inversion occurs. It’s like… I’m only prepared to take risks [using mirrors] when I know I’m going to die. (Ian)

### The self‐disgust scale

Participants total SDS‐R scores (Powell et al., 2015c) ranged from 52 up to 85 (mean 66.4, standard deviation 12.75). Eight participants rated aspects of their physical appearance as more disgusting (mean 26, standard deviation 5.72) compared to their actions and behaviours (mean 19.1, standard deviation 4.08). It is not possible to make conclusions around SDS‐R scores and participants’ demographic, mental health or suicide attempt factors due to the small sample number. However, this was not the rationale for the measure's inclusion or the aim of the current research. Instead, the SDS‐R was used to help situate the sample and triangulate the qualitative descriptions of self‐disgust with quantitative data. The measure provided additional quantitative data describing the high levels of self‐disgust across the sample. Interestingly, it was noted that participants scoring higher on the SDS‐R appeared more able to identify the traumas and stigmas that generated the ‘disgusting self’, suggesting a link between early adversities and higher levels of self‐disgust.

## DISCUSSION

This is the first qualitative study of self‐disgust in males with a history of attempted suicide. The first aim of this study was to explore whether men perceived self‐disgust to be an important emotion within their suicide attempts. It was clear that self‐disgust was a factor in the men's trajectories towards suicide from an early age. This is in keeping with the literature on the origins and the pervasive nature of self‐disgust (e.g., Powell et al., 2015b). There are similarities between the men's experiences of self‐disgust and those reported by females with depression (Powell et al., 2014), suggesting a potential commonality across these genders.

It was clear that childhood abuse was linked to a pervasive sense of the self as ‘disgusting’. These findings triangulate with the literature on trauma and self‐disgust, including feelings of contamination following abuse (see Badour & Adams, 2015; Steil et al., 2011), and the wider literature on early adversities and increased suicide risk (Fuller‐Thomson et al., 2016). Contradicting societal norms was also a source of self‐disgust in some individuals and supports the premise of self‐disgust having sociocultural elements (Powell et al., 2015a; Rozin et al., 2008). The men's self‐disgust was experienced across many aspects of the self, including their thoughts, image, feelings and behaviour. Men who had experienced multiple traumas tended to score higher on the SDS‐R, although the association between childhood adversities and SDS‐R scores remains a questions for future empirical study.

The participants differentiated self‐disgust by its embodied, visceral components (Miller, 1997; Rozin & Fallon, 1987) but for some, there were difficulties in participants’ ability to distinguish self‐disgust from other affect states, particularly shame and self‐hatred. As discussed by Power and Dalgleish (2016), shame and self‐hatred are conceptually related to disgust and it is not surprising that participants may have had some challenges in differentiating between these affective states. Furthermore, there is some evidence that disgust may become coupled with other emotions, such as anger in eating disorders (e.g., Fox et al., 2013). This may add to the challenge of differentiating affect states and it highlights the need for novel methodologies to tease these processes apart to increase our understanding.

### Self‐disgust and the process of male suicide

A second aim of this study was to understand the processes that link self‐disgust to attempted suicide for males. Self‐disgust increased suicidal risk by interacting with the endured emotions of the ‘abyss’ and feelings of worthlessness, hopelessness and disconnection. Self‐disgust appeared more pervasive in the suicide attempts of men with multiple traumas.

Disgust‐based behavioural responses, such as avoidance and recoiling, seeing oneself as repellent and contamination‐based self‐criticism all served to perpetuate the men's distress. This is consistent with the wider literature on the distancing properties of the disgust response (Powell et al., 2014, 2015b; Rozin et al., 2008) and self‐criticism's role in psychological distress (e.g., Gilbert, 2015). Interpersonal disgust has been proposed to discourage social contact (Rozin et al., 2008) and this may account for the high levels of disconnection identified in the analysis. Similarly to women with depression (Powell et al., 2014), participants reported disconnection from the self, including detachment from their reflected selves. While disconnection may help participants to cope with intense levels of self‐disgust, it paradoxically worsened the men's distress and increased their suicide risk. Exposure of men's ‘disgustingness’ was a key process in moving some men towards their attempts. For some men, this was associated with a change in mirror usage. The fear of exposure led some participants to conceal distress. Concealment has been linked to increased suicide risk (Apter et al., 2001). Future research could explore the role of disgust, exposure and concealment, as this may offer a nuance in the assessment of suicidality.

The current findings build on extant theories of suicidality. Psychological pain is central to many models of suicide (e.g., Shneidman, 1993; Williams, 1997) and the findings strongly suggested that disgust was a pronounced component of this meta‐emotional pain. Findings regarding early adversity and trauma resonate with both the Interpersonal theory (Joiner, 2005; Van Orden et al., 2010) and the Integrated Motivational Volitional (IMV) model of suicide (O’Connor, 2011). These findings also suggest the role of disconnection in exacerbating suicidal risk which resonates with the three‐step model of suicide (Klonsky & May, 2015). A crucial finding from this study highlights how self‐disgust appears to have a repelling function, where the self is repelled and distanced. This provides some support for Chu et al.’s (2013) hypothetical model of self‐disgust and suicide. However, in contrast to Chu et al.’s (2013) model, this study did not find any evidence of increasing disgust at others and the world.

### Clinical implications

This study produced some interesting findings that do translate into some pointers for clinical practice. For example, there was not extensive endorsement of perceived negative gender norms (e.g., non‐expression of emotions) as a specific risk factor for suicidality. This may be due to the inherent sampling bias within this study, as men who were not willing to share their feelings would not take part in this research study. However, the analysis did provide some nuanced links between disgust and masculinity, such as disgust's role in concealment and how the exposure of participants’ ‘disgustingness’ (i.e., abuse) may be emasculating. Although needing further research, this does appear to be an important area for assessment as this self‐disgust may lay in feelings of not being a ‘true man’ and being defective/ damaged by childhood abusive experiences. Furthermore, risk assessment should explore the nuances in which disgust may present, such as mirror usage and exposure. It was a key finding that mirror exposure was linked to increasing suicide risk for some men and this represents a simple addition to any risk assessment. Lastly, the findings from this study also highlight how self disgust may lead men to withdraw and be separate from others and providing opportunities to reconnect may potentiality reduce suicide risk (e.g., Lakeman & Fitzgerald, 2008; Struszczyk et al., 2019).

### Limitations

This study is limited by the small sample size. This was a difficult to reach population, with sampling further affected by the COVID‐19 pandemic. While first‐person narrative provides rich and valuable insight into participants’ lived experience, they are not without their limitations (see Bantjes & Swartz, 2019). Employing an interview methodology may have been impacted by various processes, such as alexithymia and memory processing difficulties at a time of heightened distress. Self‐disgust was embedded throughout the research process, including the projects promotional material and recruitment information, interview schedule and measurements used to situate the sample (i.e., SDS‐R; Powell et al., 2015c). It could be that participants were primed to think about and report this experience rather than allowing self‐disgust to emerge out of the study. It remains an empirical question as to what extent self‐disgust processes may emerge when self‐disgust is not embedded across the research process. Nevertheless, these findings are important because this is a difficult to access client group and their data are important to help further our understanding of male suicide.

## CONCLUSION

Self‐disgust appears to be an important process in male suicide risk and particularly pervasive in men with a history of multiple adversities. Interestingly, this study highlighted how self‐disgust appears within the context of emotional difficulties and mirror use. The finding that men appeared to use a mirror to confirm their self‐disgust is a potentially important avenue for future research and assessment in clinical practice.

## CONFLICTS OF INTEREST

All authors declare no conflict of interest.

## AUTHOR CONTRIBUTION

**David Mason:** Conceptualization; Data curation; Formal analysis; Writing – original draft; Writing – review & editing. **Darren James:** Methodology; Resources; Supervision; Writing – review & editing. **Liz Andrew:** Investigation; Resources; Supervision; Writing – review & editing. **John Fox:** Conceptualization; Formal analysis; Investigation; Methodology; Supervision; Writing – review & editing.

## Data Availability

The data that support the findings of this study are available upon request from the corresponding author. The data are not publicly available due to privacy or ethical restrictions.

## APPENDIX A

### Suicide questionnaire created by the authors

**These next questions relate to your experience of suicide attempts. We appreciate that some of these questions may be very difficult to answer, however we would be grateful if you could try to answer each question. Please speak to the researcher if you have any concerns or questions**.

When did you last attempt to kill yourself?

**Table:**

0–6 Months ago	
6–12 Months ago	
12–18 Months ago	
18–24 Months ago	
Over 24 Months ago	

During your most recent attempt to kill yourself, did you want to die?

**Table:**

I attempted to kill myself, but did not want to die.	
I attempted to kill myself, and really wanted to die.	

During your *most recent* attempt to kill yourself, did you leave a suicide note?

**Table:**

No	
I thought about writing a note but didn't	
I wrote a note although tore it up/threw it away	
Yes	

During your *most recent* suicide attempt, did you tell anybody, or did anybody find you?

**Table:**

I told someone I was planning to kill myself	
I told someone after I had acted on my plans to kill myself (for example taken an overdose and then contacted somebody)	
Somebody disturbed me/found me	
None of the above	
Other _______________	

Did you require medical treatment as a result of your *most recent* attempt to kill yourself?

**Table:**

I woke up in hospital	
I took myself to hospital	
I called an ambulance	
I was conscious and somebody else called an ambulance	
No medical treatment was accessed.	
I informed my GP some time after	
Other _________________________________	

We appreciate that these questions can be very difficult to answer and thank you for taking the time to complete this form. In the box below, we would be grateful if you would be able to describe the method that you used in your most recent suicide attempt and any other information you feel may be relevant to your *most recent* suicide attempt.

Prior to your most recent attempt to kill yourself, how many previous attempts have you made?

**Table:**

None	
One	
Two	
Three	
Four or More	

**Thank** **you very much for taking the time to fill out this questionnaire. If you have any questions, please ask a member of the research team**.

## APPENDIX B

### Final interview schedule used

**The** **role of self‐directed disgust in males who have attempted suicide – Interview guide**

*Before commencing the interview, revisit confidentiality and ensure the consent form is signed and the participant has read the participant information sheet. Participants are reminded that the interview is being recorded and transcribed and that they have the right to withdraw*.

Introduction:

*At the start of the interview, begin with: Thank you for taking part in this interview. The questions will be looking at your personal experiences and understanding of disgust directed towards the self, or self‐disgust, and whether this is associated with suicide. I understand that the topic being discussed is very sensitive, therefore if you would like to stop the interview at any time please let me know. If you need to stop the interview and take a break then that is fine, please just let me know. Also, if you have any concerns during the interview please let me know. Do you have any questions before we begin?*

1) During this interview, I will ask you some questions about what you were thinking and feeling at a particular time. Do you find it easy to know what you are feeling?

These don’t have to be big events, but…

a. Can you give me an example of a time when you've felt angry?

b. Can you give me an example of a time when you've felt sad?

c. Can you give me an example of a time when you've felt worried?

d. Could you give me some examples about how your body reacts to certain feelings (for example, people might smile when they feel happy)?

2) When did you first experience suicidal thoughts?

***Prompt*** – What were you thinking then?
***Prompt*** – What feelings come up when you have suicidal thoughts?

3) Could you please tell me about your most recent suicide attempt?

***Probe* –** What was going on in your life then?
***Probe –*** What were you feeling then?
***Probe* –** Could you describe the events that led up to your attempt?
***Prompt –*** What was going on for you in the days, weeks and months leading up to this attempt?

4) How would you describe the person that you were then?

***Probe –*** How would you describe the person you are now?

5) Could you please tell me about any other suicide attempts?

***Probe* –** What was going on in your life then?
***Probe –*** What were you feeling then?
***Probe*** – Could you describe the events that led up these attempts?
***Prompt –*** What was going on for you in the days, weeks and months leading up to these attempts?

6) What is your understanding of the word disgust (or revulsion)?

***Probe –*** What words, phrases and images pop into your head when you think about disgust or when I just asked you that question?

7) What do you do when you find something disgusting?

***Probe –*** Can you give me an example of a time you’ve felt disgusted with something or someone?
***Probe –*** What did you do?
***Probe –*** What did you notice going on in your body when you thought of that?

8) What is your understanding of the emotion of self‐disgust?

***Probe –*** What words, phrases and images pop into your head when you think about self‐disgust or when I just asked you that question?

9) Do you ever feel disgusted with yourself?

***Prompt –*** When, if at all, did you first experience self‐disgust?
***Prompt –*** Can you describe a time when you have felt disgusted with yourself?
***Probe –*** What do you do when you feel like that?

10) What are you able to do with feelings of self‐disgust when they arise?

***Probe*** – In which way are these similar, or different, to what you’re able to do with suicidal feelings and thoughts when they arise.
***Probe*** – Has it always been like this?
***Probe*** – Some people interviewed for this project mentioned that they drank alcohol or used drugs to manage feelings of self‐disgust. Has this ever applied to you?

11) As you look back on your suicide attempt(s), do you think that any thoughts, images or feelings of self‐disgust were present at that time?

***Probe –*** What were they like?
***Probe –*** Did they seem stronger than any other thoughts or feelings?
***Probe –*** What relationship did they have to your suicide attempt?

12) Has your experience of self‐disgust changed since your suicide attempt?

***Probe* – **In what way?

13) Do you think self‐disgust is different to other emotions such as self‐hatred, self‐dislike, shame and guilt? In what way?

***Probe –*** Can you tell me of a time when you felt shame?
***Probe –*** Do you think, and in what way, is shame different to self‐disgust?
***Probe –*** Are you able to notice the difference between times when you feel ashamed and times when you feel self‐disgust?
***Prompt –*** What helps you to distinguish between these.

14) The next questions relate to some common areas which have come out of other people interviewed for this project. As before, you can choose not to answer any questions if you don't want to. I’m about to ask a question about historic abuse; if you do decide to answer this, please be assured that I will not ask you to elaborate any further on this and won't ask any details, unless you mention anything that raises concerns about your current safety.
  a. Did you experience sexual abuse, either as a child or an adult?
  b. Did you experience any other adversities as a child (such as neglect, physical abuse, emotional abuse, bullying)?

**OMIT** **THIS QUESTION IF TOPIC HAS SPONTANEOUSLY ARISEN DURING INTERVIEW**

15) Would you be able to tell me about your relationship with mirrors?

***Probe –*** Do you notice any difference in how you use mirrors when you feel self‐disgust?
***Probe –*** Do you notice any difference in how you use mirrors when you experience suicidal thoughts or feelings?

16) Would you be able to tell me about your relationship with food?

***Probe –*** Do you notice any difference about your relationship with food when you feel self‐disgust?
***Probe –*** Do you notice any difference about your relationship with food when you experience suicidal thoughts or feelings?

Closure Questions to attempt to bring mood close to baseline

17) Is there something that you might not have thought about before that occurred to you during this interview?
18) What has it been like answering these questions?
19) Have you learned anything about yourself since your suicide attempt?
20) Is there something else you think I should know to understand self‐disgust and suicide, or male suicide better?

Close, thank for time and move to debrief

General prompts

In what way?

Can you tell me more?

What did you do then?

Can you explain a bit more about that?"

"can you explain what you mean?"

Is there anything else about…?

Are you able to give me an example?

what thoughts were you having then/now?

What were you feeling then/now?

What is it you are feeling?

What is the…[e.g. missing ingredient]

What feelings are attached to these thoughts?

Can you tell me more about those feelings of….

What are those feelings of….like?

Can you tell me in your own words
